
JOURNAL INFORMATION
==============================
NLM Title Abbreviation: Inter Econ
Journal ID: Inter Econ
NLM Journal ID: 101086399

Inter Economics

ISSN: 0020-5346

ARTICLE INFORMATION
==============================
PMCID: PMC7385196
DOI: 10.1007/s10272-020-0901-5
Article ID: 901
Article version: 1
Subjects: Forum Introduction

Reconstructing the EU After COVID-19

Electronic publication date: 2020 Jul 28
Print publication date: 2020
Volume: 55
Issue: 4
First page: 204
Last page: 204
Copyright: © The Author(s) 2020
License: Open Access This article is licensed under a Creative Commons Attribution 4.0 International License, which permits use, sharing, adaptation, distribution and reproduction in any medium or format, as long as you give appropriate credit to the original author(s) and the source, provide a link to the Creative Commons licence, and indicate if changes were made.
The images or other third party material in this article are included in the article’s Creative Commons licence, unless indicated otherwise in a credit line to the material. If material is not included in the article’s Creative Commons licence and your intended use is not permitted by statutory regulation or exceeds the permitted use, you will need to obtain permission directly from the copyright holder.
To view a copy of this licence, visit http://creativecommons.org/licenses/by/4.0/.
License URL: https://creativecommons.org/licenses/by/4.0/

issue-copyright-statement: © The Author(s) 2020

==============================
DOI: 10.1007/s10272-020-0901-5

### Reconstructing the EU After COVID-19

**On 21 July 2020, EU leaders announced a ground-breaking and highly anticipated plan, the 'Next Generation EU', to jointly borrow €750 billion to respond to the coronavirus pandemic. The EU's new recovery plan, which will be composed of grants and loans attached to the new Multiannual Financial Framework, is the culmination of what many consider a painfully slow and uncoordinated European response to the COVID-19 crisis and the far-reaching effects it has had on the European economy. While a crisis of this magnitude could threaten the EU's very existence, deepening divides and sewing the seeds of anti-European sentiment, it may conversely also be an opportunity for the EU to address some of the fundamental flaws that exist in the euro area architecture. The pandemic response is a chance for Europe to reboot by focusing on a number of mechanisms at hand, including state aid, taxation and labour reallocation, among others. The EU should not lose sight of broader societal goals such as climate neutrality and digitisation but rather prioritise them. Ultimately, if handled correctly, the coronavirus pandemic has the potential to bring the EU together like no other event since its foundation.**

### European Pandemic Recovery: An Opportunity to Reboot

**Agnès Bénassy-Quéré**, Université Paris 1 Panthéon-Sorbonne, France.

**Beatrice Weder di Mauro**, Johannes Gutenberg University of Mainz, Germany.

### A Framework for a European Economic Recovery After COVID-19

**Julia Anderson**, Bruegel, Brussels, Belgium.

**Simone Tagliapietra**, Bruegel, Brussels, Belgium.

**Guntram B. Wolff**, Bruegel, Brussels, Belgium.

### Common Fiscal Capacity Is Needed to Strengthen Risk Sharing

**Antonia Díaz**, Universidad Carlos III de Madrid, Spain.

### State Aid Policies in Response to the COVID-19 Shock: Observations and Guiding Principles

**Massimo Motta**, ICREA – Universitat Pompeu Fabra, Barcelona; and Barcelona Graduate School of Economics, Spain.

**Martin Peitz**, University of Mannheim; and Mannheim Centre for Competition and Innovation, Germany.
